# Draft genome of *Brugia pahangi*: high similarity between *B. pahangi* and *B. malayi*

**DOI:** 10.1186/s13071-015-1064-2

**Published:** 2015-09-08

**Authors:** Yee-Ling Lau, Wenn-Chyau Lee, Jinquan Xia, GuiPing Zhang, Rozaimi Razali, Arif Anwar, Mun-Yik Fong

**Affiliations:** Department of Parasitology, Faculty of Medicine, University of Malaya, 50603 Kuala Lumpur, Malaysia; Singapore Immunology Network (SIgN), Agency for Science, Technology and Research (A*STAR), Singapore, 138648 Singapore; BGI-Shenzhen, Shenzhen, 518083 China; Sengenics HIR, University of Malaya, 50603 Kuala Lumpur, Malaysia

**Keywords:** *Brugia pahangi*, *Brugia malayi*, Draft genome

## Abstract

**Background:**

Efforts to completely eradicate lymphatic filariasis from human population may be challenged by the emergence of *Brugia pahangi* as another zoonotic lymphatic filarial nematode. In this report, a genomic study was conducted to understand this species at molecular level.

**Methods:**

After blood meal on a *B. pahangi*-harbouring cat, the *Aedes togoi* mosquitoes were maintained to harvest infective third stage larvae, which were then injected into male Mongolian gerbils. Subsequently, adult *B. pahangi* were obtained from the infected gerbil for genomic DNA extraction. Sequencing and subsequently, construction of genomic libraries were performed. This was followed by genomic analyses and gene annotation analysis. By using archived protein sequences of *B. malayi* and a few other nematodes, clustering of gene orthologs and phylogenetics were conducted.

**Results:**

A total of 9687 coding genes were predicted. The genome of *B. pahangi* shared high similarity to that *B. malayi* genome, particularly genes annotated to fundamental processes. Nevertheless, 166 genes were considered to be unique to *B. pahangi*, which may be responsible for the distinct properties of *B. pahangi* as compared to other filarial nematodes. In addition, 803 genes were deduced to be derived from *Wolbachia*, an endosymbiont bacterium, with 44 of these genes intercalate into the nematode genome.

**Conclusions:**

The reporting of *B. pahangi* draft genome contributes to genomic archive. Albeit with high similarity to *B. malayi* genome, the *B. pahangi*-unique genes found in this study may serve as new focus to study differences in virulence, vector selection and host adaptability among different *Brugia* spp.

**Electronic supplementary material:**

The online version of this article (doi:10.1186/s13071-015-1064-2) contains supplementary material, which is available to authorized users.

## Background

Lymphatic filariasis, which is also known as elephantiasis tropica, affects more than 120 million people worldwide [1]. This immune-mediated disease is typically caused by the presence of filarioid nematodes (e.g., *Wuchereria bancrofti*, *Brugia malayi*, and *Brugia timori*) [2]. These filarial nematodes are transmitted to humans via mosquitoes from the genera *Mansonia, Anopheles*, *Culex*, and *Aedes*. Lymphatic filariasis causes enlargement of body parts and skin exfoliation, which can result in severe disability, disfigurement, and social stigma [3]. The most common etiological agent of lymphatic filariasis is *W. bancrofti* [2]. Nevertheless, certain *Brugia* spp. have been found to routinely cause filariasis in various Asian countries including India, Malaysia, Indonesia, the Philippines, and China [4]*. W. bancrofti*, *B. malayi* (nocturnal periodic strain, whose microfilaria can only be detected from peripheral circulation between dusk and midnight), and *B. timori* exclusively infect humans, whereas the nocturnal subperiodic strain of *B. malayi* (whose microfilaria can be found in peripheral circulation throughout the day, but peak between noon and dusk) is zoonotic and transmitted to humans (via mosquitoes) from cats, dogs, and wild carnivores [4, 5]. Although both the aforementioned *Brugia* spp. commonly infect humans, recent evidence has also indicated that *Brugia pahangi*, a filarial nematode that is naturally found in cats, can cause clinical infection in humans, with clinical presentations that are consistent with lymphatic filariasis [6]. This suggests that *B. pahangi* infection may be more prevalent in humans than previously thought. Nevertheless, it is known that the physiology and vector competence of *B. pahangi* and *B. malayi* differ from each other [5, 7, 8]. Clearly, understanding the relationships of distinct nematodes with their invertebrate vectors, as well as humans and other definitive hosts, has major implications for the development of effective filariasis control and eradication strategies. With advances in science and technology, genomic analyses provide a rapid and efficient way to decipher genetic structure, function, and relationships. Here, we have characterized the genome of *B. pahangi* and compared it with those of other nematodes in order to deduce biological similarities and differences among these nematodes.

## Methods

### Genomic sequencing and pre-processing of reads

Female *Aedes togoi* mosquitoes were fed on a cat naturally infected with *B. pahangi*. The infective third stage larvale (L3) of *B. pahangi* were recovered from the infected mosquitoes. Subsequently, 100 L3 larvae suspended in 0.5 ml RPMI were injected into each of the recruited male Mongolian gerbils (*Meriones unguiculatus*) via intra peritoneal and subcutaneous routes of administration. Sluggish and inactive larvae were not used. Three months later, thick blood smears of the gerbils were made and stained with Giemsa for microfilariae detection [9]. At 95 days post infection, gerbils were sacrificed for recovery of adult worms using protocols as described previously [10, 11]. The adult worms were then examined under the microscope. Approval for using gerbils in our study was granted by the University of Malaya Animal Care and Use Committee (Ref. No. PAR/29/06/2012/RM [R]).

Female worms were collected and washed 3 times with sterile physiological saline before genomic DNA extraction. High molecular weight genomic DNA was isolated from a single female worm of *B. pahangi* using DNeasy Blood and Tissue Kit (QIAGEN, Germany). The specific identity of the worm was verified by PCR amplification using primer pairs specific for the cytochrome oxidase I (COXI) gene of *B. pahangi* (forward primer 5' TATTGCCTGTTATGC 3', reverse primer 5' TGTATATGTGATGAC 3') and DNA sequencing (Reference GenBank accession no. AJ271611) [12]. The DNA yield was measured spectrophotometrically (*Qubit* fluorometer dsDNA HS Kit, Invitrogen); DNA integrity was verified by agarose gel electrophoresis and using a Bioanalyzer (2100, Agilent).

Paired-end (PE) genomic libraries (with inserts of 170 bp, 500 bp and 800 bp) and Jumping (J) genomic libraries (with inserts of 2 kb, 5 kb, and 10 kb) were constructed [13, 14]. To produce sufficient amounts of DNA for these libraries, 250-500 ng of genomic DNA were subjected to whole genome amplification (WGA) using the REPLI-g midi kit (Qiagen). Sequencing was carried out on GA II or HiSeq (Illumina; 2×75 or 2×100 reads for paired-end libraries, and 2×49 reads for jumping libraries). Reads were exported to FASTQ format [15]. Sequencing was carried out to a total amount of 24 GB, which is equivalent to 214 fold coverage of the whole genome (Additional file 1: Table S1). To minimize sequencing errors, the following were filtered: artificial reads, redundant reads, reads with adapter length of ≥ 10 bp and a mismatch rate ≤ 0.1, reads with > 2 % ambiguous bases or poly-A, reads with low-quality bases of < 40 PHRED score value for 170-800 bp libraries and < 60 PHRED for 2 to 10 kb libraries, short reads where 2 reads for the paired ends overlapped ≥ 10 bp with mismatch of lower than 10 %, PCR duplicate reads, as well as reads with adapter contamination. Genomic reads from individual libraries were assessed for quality control, adaptors removal and sequencing errors correction using an in-house pipeline. *Mus muculus* and *Wolbachia* of *B. malayi* (accession number AE017321) were identified by comparison to the ENSEMBL (release 72) and NCBI (build 37) databases respectively. To do this, all libraries were aligned to the *Mus musculus* genome using SOAPaligner v.2.21 [16], and reads that mapped to this genome were removed. To remove bacterial sequences, reads from each library were aligned (≥80 % of read length and ≥ 40 % sequence identity in overlapping regions) to known *Wolbachia* genomes using BLAST v.2.2.26 [17, 18].

### Genome assembly

Pre-processed genomic PE-libraries and J-libraries were assembled and scaffolded using the program SOAPdenovo with k-mer of 29 [18]. During assembly, de Bruijn graph was used to assemble all possible sequences from the Illumina reads, with a k-mer as a node and the k-1 bases overlap between 2 k-mers as an edge. In the assembly process, the tips and k-mers with low coverage in the graph were eliminated to reduce sequencing errors and limited branches. The graph was converted to a contig graph by transforming linearly connected k-mers into a pre-contig node. The Dijkstra’s algorithm (Skiena) was used to detect bubbles, which were then merged into a single pathway when the branches sequences were identical. Using this method, consensus sequences were obtained. Contigs were linked to a scaffolding graph with PE reads. Connections between contigs comprised the edges in this graph and the branch length demonstrated the gap size, which was calculated from the insert size of the PE reads. After that, sub-graph linearization was applied to transform interleaving contigs into a linear structure and repeat masking was used to mask complicated connections for repeat contigs. Using this approach, contigs in any non-linear structure could be considered compatible. Subsequently, PE reads were applied in step-by-step manner, with increasing insert sizes of 170 bp, 500 bp, 800 bp, 2 kb, 5 kb and 10 kb respectively. Subsequently, gaps between contigs were filled. The GC-depth content was examined to analyse nucleotide distribution, randomness of sequencing and inspect for possible sample contamination. We used 10 kb non-overlapping sliding windows and calculated the GC content and average depth among the windows. Syntenic blocks between the genome of *B. pahangi* and *B. malayi* and other genomes were detected using the program LASTZ [19]. A pairwise whole-genome alignment was conducted using the following settings: T = 2 (no transition), C (chain) = 2, H (inner) = 2000, Y (y drop) = 3400, L (gapped thresh) = 6000 and K (hsp thresh) = 2200. ChainNet, which can accommodate inversions, translocations, duplications, large-scale deletions, and overlapping deletions, was used to combine traditional alignments into large structures.

### Prediction of repetitive elements

Tandem repeat elements (TEs) were identified using the software Tandem Repeat Finder (4.04) with the following settings: Match = 2, Mismatching penalty = 7, Delta = 7, PM = 80, PI = 10, Minscore = 50, MaxPeriod = 2000. TEs were identified using homology and *de novo* approaches. The homology approach used standard databases containing known repetitive sequences (Repbase), and predicted TEs at both the DNA and protein levels. For the former, RepeatMasker was applied [20], together with the Repbase library; for the latter, RepeatProteinMask was used to compare against the TE protein database in Repbase using the program RMblast2.0 [21, 22]. The *de novo* approach employed LTR_FINDER [22], PILER [23] and REPEATSCOUT (Skiena). The results from LTR_FINDER [22], PILER [24] and REPEATSCOUT (Skiena) were merged into a library, which was then used by RepeatMasker [24] to identify homologous repeats in the draft genome assembly for *B. pahangi*, and to categorize them (Additional file 1: Table S14).

### Prediction of the gene set and annotation

To predict gene structures in the draft genome assembly, we performed both *de novo*- and homology-based predictions. For homolog predictions, we used protein sets of *B. pahangi*, *B. malayi, Caenorhabditis briggsae*, *Caenorhabditis elegans*, *and Pristionchus pacificus* from ENSEMBL [25], and mapped them to the assembled genome using TBLASTN2.2.23 using an E-value of 10^-5^. After that, we selected the most homologous protein for each genomic locus showing multiple matches. Subsequently, we removed regions with < 25 % homology of the query protein. We extended 500 bp at both ends of the alignment regions and predicted gene structures using the program GeneWise2.2.0 [26]. For *de novo* predictions, we used the programs AUGUSTUS [27], SNAP [28] and GLIMMERHMM [29]. Genes with coding length < 150 bp were eliminated to reduce false positives. Subsequently we used GLEAN2.2 to integrate predictions, in order to generate a consensus gene set [30]. Functions were assigned to individual genes according to the best alignment match using BlastP to SwissProt [31] and TrEMBL [32] databases. The motifs and domains of genes were determined using InterProScan [33] against protein databases Pfam [34], PRINTS [35], PROSITE [36], ProDom [37] and SMART [38]. Gene Ontology (GO) identities of individual genes were obtained from the corresponding InterPro entry [39, 40]. Each gene was assessed for a known functional orthologue using the Kyoto Encyclopaedia of Genes and Genomes (KEGG) and orthologous matches were mapped visually to a defined pathway using the KEGG pathway tool [41]. Functional annotation of the unique *B. pahangi* genes has revealed key data that can further our understanding of differences among different filarial species. A number of genes and pathways of interest were selected for case study analysis. In this regard, we used BlastP against GO [39] and KEGG databases [41]. The genes were then filtered (sequence identity >30 %; sequence length coverage >80 %) and ranked according to their E-values. Apart from analytical and functional comparison, whole genome synteny analysis was also performed to identify and characterize functional related stretches of genes that are clustered. LASTZ was used to perform the pairwise whole-genome alignment and coverage is calculated by number of nucleotides divided by the total length of the provided intervals

### Clustering of gene orthologs

All protein sequences were compared against database containing protein dataset of all species covered in this study (BlastP; using an E-value < 10^-7^), and con-joined fragment alignments for every gene were used using the program Solar (Skiena) when necessary. We assigned a connection (edge) between 2 nodes (genes) if more than 1/3 of the region aligned to both genes. H score (0 to 100) was used to weight similarity (edge) between the nodes. For two genes G1 and G2, the H score was defined as score (G1G2)/max (score (G1G1), score (G2G2), the score used here was the BLAST raw score. Extracting gene families using clustering by Hcluster_sg [42], we used the average distance for the hierarchical clustering algorithm, which required the minimum edge weight (H score) to be larger than 5, and the minimum edge density (total number of edges/theoretical number of edges) to be larger than 1/3. The clustering of gene families was terminated if 1 or more outgroup genes had been identified.

### Phylogenetic analysis

We constructed a phylogenetic tree of *B. pahangi* and other related genomes using 1673 single-copy orthologuous genes. Genes of *Drosophila melanogaster* was used as outgroup. Blast TreeView with Neighbour Joining method (maximal allowed fraction of mismatched bases = 0.6) based on Kimura model was applied [43]. Similarly a separate phylogenetic tree was constructed from the *Wolbachia* genes.

### Data access

This Whole Genome Shotgun project has been deposited at DDBJ/EMBL/GenBank under the accession JRWH00000000 (BioProject number, PRJNA263436; BioSample, SAMN03100407). The version described in this paper is version JRWH01000000.

## Results

**Fig. 1:**
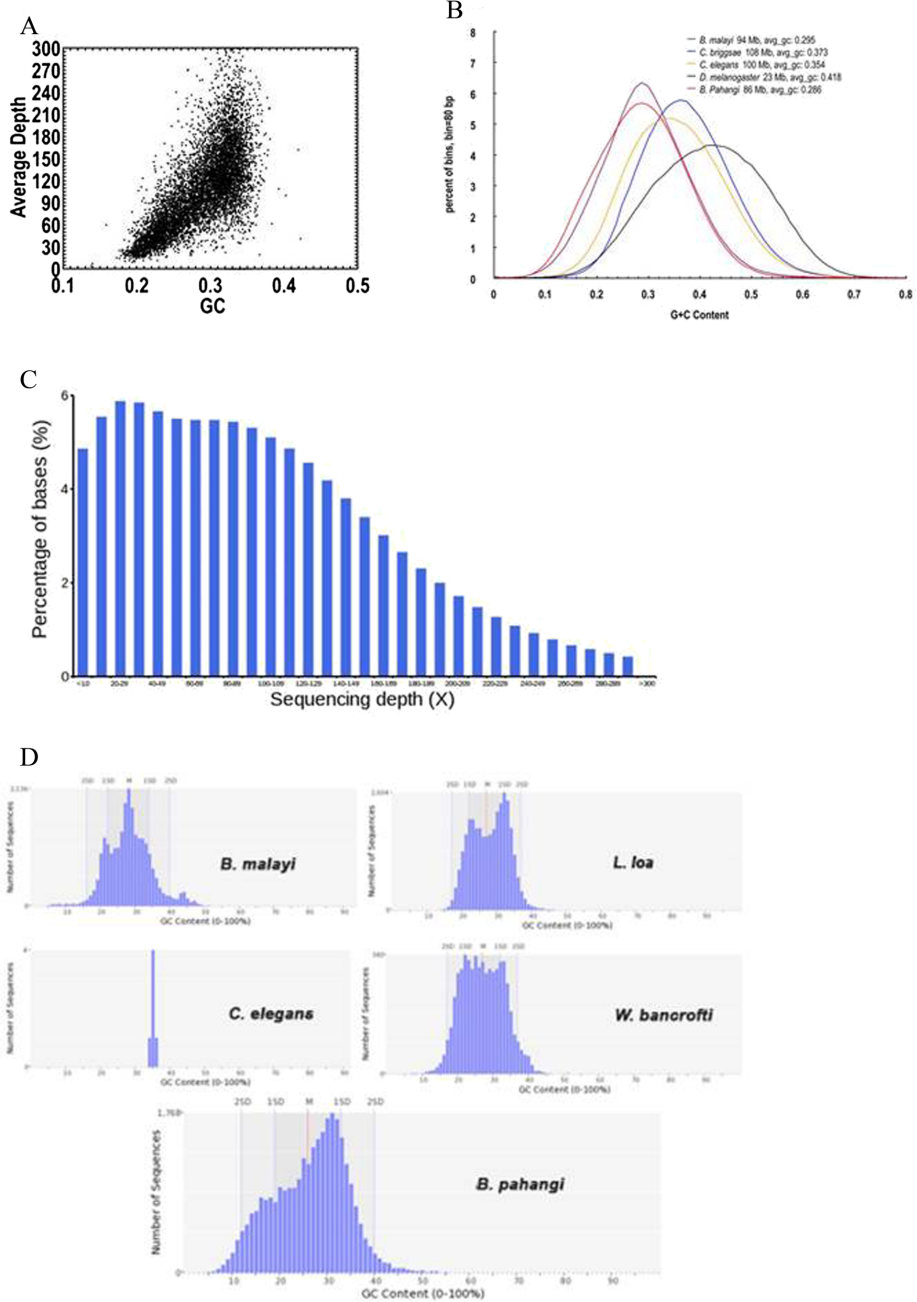
**a** Plot showing content and sequencing depth. The GC content for *B. pahangi* is high at the average depth of in between 10 to 180 with GC content value in range of 0.2 to 0.4. **b** Graph showing GC content distributions within genomes of different species under study. We used 500 bp bins (with 250 bp overlap) sliding along the genome. The highest percent of bins for *B. pahangi* is in between 5 to 6 % with GC content of 0.3. **c** Graph showing sequence depth distribution. The filtered reads were aligned onto the assembly genome sequence using SOAP. The percentage of bases is increasing from the starting point up till sequencing depth of 20-29 with optimal percentage of bases of 6 %, and decreases from this point until the sequencing depth of 60-69. From that point, the percentage of bases is stable until sequencing point 80-89 (before falling dramatically). **d** Plots showing comparison of GC content between different genomes

We sequenced the *B. pahangi* genome at 168-fold coverage (Additional file 1: Tables S1 and S2) and produced a final draft assembly of 85.4 Mb and 1.4 Mb of bacterial (*Wolbachia*) chromosomal genome sequences (N50 = 155.8 kb; 29,435 scaffolds) (Table 1). The mean GC-content of the *B. pahangi* genome was 28.5 % (Fig. 1). A Core Eukaryotic Genes Mapping Approach (CEGMA) score of 94.32 % was detected. The generated draft genome was estimated to contain a repeat content of 5.9 % (~4.9 Mb of DNA), which includes 0.924 % DNA transposons, 0.466 % LINE, 0.009 % SINE, 0.774 % LTR, 0.917 % unclassified dispersed elements and 0.008 % other elements (Additional file 1: Table S3). A total of 476,584 LTR, 369,943 LINE, and 7123 SINE were identified across the whole draft genome (Additional file 1: Table S3). In addition, a total of 9687 protein-encoding genes were predicted. The average sizes of exons and introns were found to be 153 bp and 345 bp respectively, with an average of 5.9 exons per gene (Additional file 1: Table S4).

**Table 1:** Comparison of assembly statistics

	*B. pahangi*	*B. malayi*	*C. elegans*	*W. bancrofti*	*L. loa*	*wBp*	*wBm*	*wWb*
Coverage	168x	9x	-	12x	20x	168x	11x	2x
Sequence (Mb)	85.4	93.7	100	81.5	91.4	1.4	1.08	1.05
#Scaffolds	29,435	24,285	-	25,884	5,774	227	1	763
Scaffolds N50 (kb)	155.8	94	-	5.16	172	933.1	1,080	1.62
GC (%)	28.54	30.2	-	29.7	31.0	34.14	34.18	34.0
Genes (n)	9,687	18,348	-	19,327	14,907	803	805	-
# reads	29,569	24,285	6	5,774	25,884	227	1	763
Length distribution								
Mean (bp)	2,900	3,856	16,712,035	15,825	3,149	5,444	1,080,084	137,9.20
Minimum (bp)	100	200	13,783,682	265	500	100	1,080,084	503
Maximum (bp)	1,247,913	6,534,162	20,924,143	1,325,655	62,423	933,146	1,080,084	16,892
Mode (bp)	101 with 393 sequences	908 with 58 sequences	13,783,682 with 1 sequence	586 with 13 sequences	561 with 25 sequences			-
GC Distribution								
Mean (%)	28.54	28.13	35.00	27.04	27.84	34.14	34.18	-
Minimum (%)	0.01	0	34	7	9	0.13	34.18	-
Maximum (%)	60.18	72	36	51	60	43.9	34.18	-
Mode (%)	31 with 1,768 sequences	28 with 2,133 sequences	35 with 4 sequences	22 with 340 sequences	32 with 2,004 sequences			-
Ambigous Base (N)								
# of sequences with N	883 (2.99 %)	1,011 (4.16 %)	0 (0 %)	1,160 (20.09 %)	1,048 (4.05 %)	5 (2.2 %)	0	-
Assembly quality measure								
N50	152,403	41,387	17,493,784	174,388	5,161	933,146	1,080,084	-
N90	1,249	965	13,783,682	18,595	1,343	26,271	1,080,084	-
N95	463	842	-	2,078	956	-	-	-

*wBp*, *Wolbachia* of *B. pahangi*; *wBm*, *Wolbachia* of *B. malayi*; *wWb*, *Wolbachia* of *W. bancrofti*

**Fig. 2:**
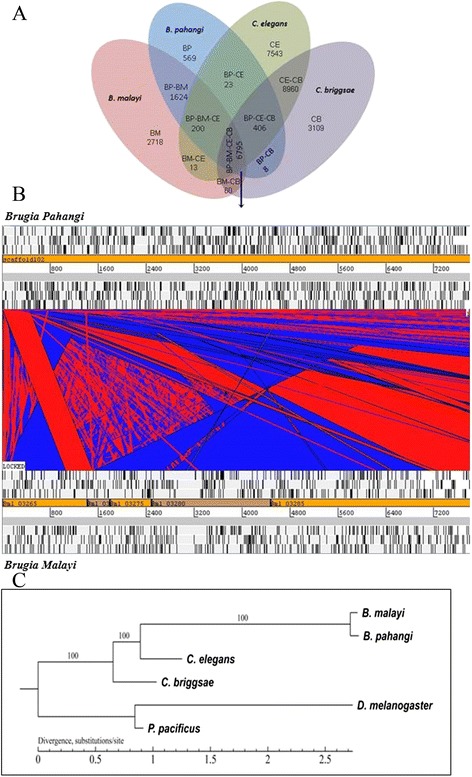
**a** Venn diagram showing the overlapping genes between *B. pahangi* and other similar species prior to filtering of *Wolbachia* genes. **b** Relative arrangements of *B. pahangi* genes and their orthologs on *B. malayi*. Forward and reverse strands are distinguished based on position (i.e., forward on top and reverse below). **c** A phylogenetic tree constructed based on *B. pahangi* and other sequenced genomes using single-copy orthologous genes. The different molecular clocks (i.e., divergence rates) might be explained by the body size or generation time hypotheses, which suggest that larger body size or longer generation time result in a slower molecular clock

Comparison between draft genome sequences of *B. pahangi* and those of *B. malayi*, *C. briggsae* [44], and *C. elegans* [45] was performed (Additional file 1: Table S4). We found that *B. pahangi* genome shows the highest sequence similarity to those of *B. malayi*. From the predicted *B. pahangi* genes, 90 % (8681 genes) appears to have orthologs (BLASTp cut-off: 10^-5^) in *B. malayi*, as compared to that in *C. elegans* (n = 7424; 77 %) and *C. briggsae* (n = 7271; 75 %). Overall, 6795 genes were found to be orthologous among all 4 species under comparison. Another 62 predicted *B. pahangi* genes were found to be shared among *B. malayi* and *C. briggsae*, and 1624 *B. pahangi* genes were shared exclusively with *B. malayi*. A total of 569 genes were predicted to be unique to *B. pahangi* (Fig. 2a). Via whole genome conserved synteny analysis using LASTZ pairwise genome alignment [19], we observed high rates of agreement upon comparison between *B. pahangi* and *B. malayi* genomes, with a genome coverage range of 70-75 % (Fig. 2b).

A total of 7801 out of the 9687 predicted *B. pahangi* genes (~80.5 %; Additional file 1: Table S5) were annotated in the Swiss-Prot (6933 genes; 71.57 %; Additional file 1: Table S6), InterProScan (7201 genes; 74.33 %; Additional file 1: Table S7), GO (5837, 60.27 %; Additional file 1: Table S8) KEGG (5780 genes; 59.67 %; Additional file 1: Table S9) databases. Most of the 7201 InterProScan-annotated genes were found in other databases, namely PFAM (6769 genes; 94.00 %), PANTHER (6469 genes; 89.83 %), FPrintScan (1,443 genes; 20.03 %), ProDom (104 genes; 1.44 %) and SMART (3081 genes, 42.79 %). On the other hand, for KEGG annotation, the predicted proteins encompass peptidases (n = 105), kinases (n = 425), phosphatases (n = 230), transporters (n = 10) and CTPases (n = 2), with some of the proteins having multiple functions (Additional file 1: Table S6). For the KEGG-mapped genes, 4927 genes (~50.86 % of the total predicted *B. pahangi* genes) coded for known proteins involved in various cellular pathways, and another 853 genes (~8.8 % of the total predicted genes) encoded for proteins of mostly unknown functions (i.e. orphans). These KEGG-mapped genes were further divided into 6 different function classes, namely cellular pathways (1118 genes; ~23 %), environmental information processes (1129 genes; ~23 %), genetic information processing (2351 genes; ~48 %), metabolism (2001 genes; ~41 %) and organismal systems (870 genes; ~18 %).

To find out the shared and conserved elements encoded in the *Brugia* genome, comparative analyses of the functional annotation in genes shared between *B. pahangi* and other nematodes were performed. KEGG database was used to examine the 6795 genes shared among *B. pahangi*, *B. malayi*, *C. elegens* and *C. briggsae*. Of these, 5140 (~75.64 %) could be mapped to biological pathways. Of these mapped genes, 5131 (~99.82 %) were coded for known proteins whereas 9 genes (~0.18 %) encoded for orphan proteins. The encoded known proteins encompassed a number of functional classes, namely genetic information processing (2056 genes involved, ~40.07 %), metabolism (1768 genes involved; ~34.46 %), environmental information processing (1046 genes involved, ~20.39 %), cellular processes (1021 genes involved; ~19.90 %), and organismal systems (810 genes involved; ~15.79 %). Interestingly, comparison between *B. pahangi* and *B. malayi* revealed that the shared gene set was closely associated with phosphorylation activity. Most of the *Brugia* spp. conserved genes are coded for fundamental and common biological processes such as oxidation-reduction transports, DNA replication machinery and transcription regulation. As expected, comparisons of *B. pahangi* genome with those of *Caenorhabditis* spp. reflected larger differences. For instance, GTPase-mediated signal transduction machinery could be found in both species of *Caenorhabditis* spp. studied. However, this signal transduction machinery was not available in *B. pahangi* genome. On the other hand, protein histidine kinase activity was encoded by *B. pahangi* genes, but not available in *C. briggsae* and *C. elegans*.

From the analysis, 569 genes were predicted to be unique to *B. pahangi* (Additional file 1: Table S10). However, 403 of these genes were found to be originated from endosymbiotic bacterium *Wolbachia* (Additional file 1: Table S11). For the remaining 166 non-*Wolbachia*, *B. pahangi* unique genes, 26 genes were mapped to known KEGG pathways (Additional file 1: Table S12). Many of these genes were mapped to multiple functions, which include genetic information processing (10 genes involved), metabolic processes (9 genes involved), cellular processes (8 genes involved), environmental information processing (5 genes involved), organismal systems (4 genes involved), and human disease pathways (3 genes involved). These genes are responsible for expressions that may make *B. pahangi* a distinct species.

**Fig. 3:**
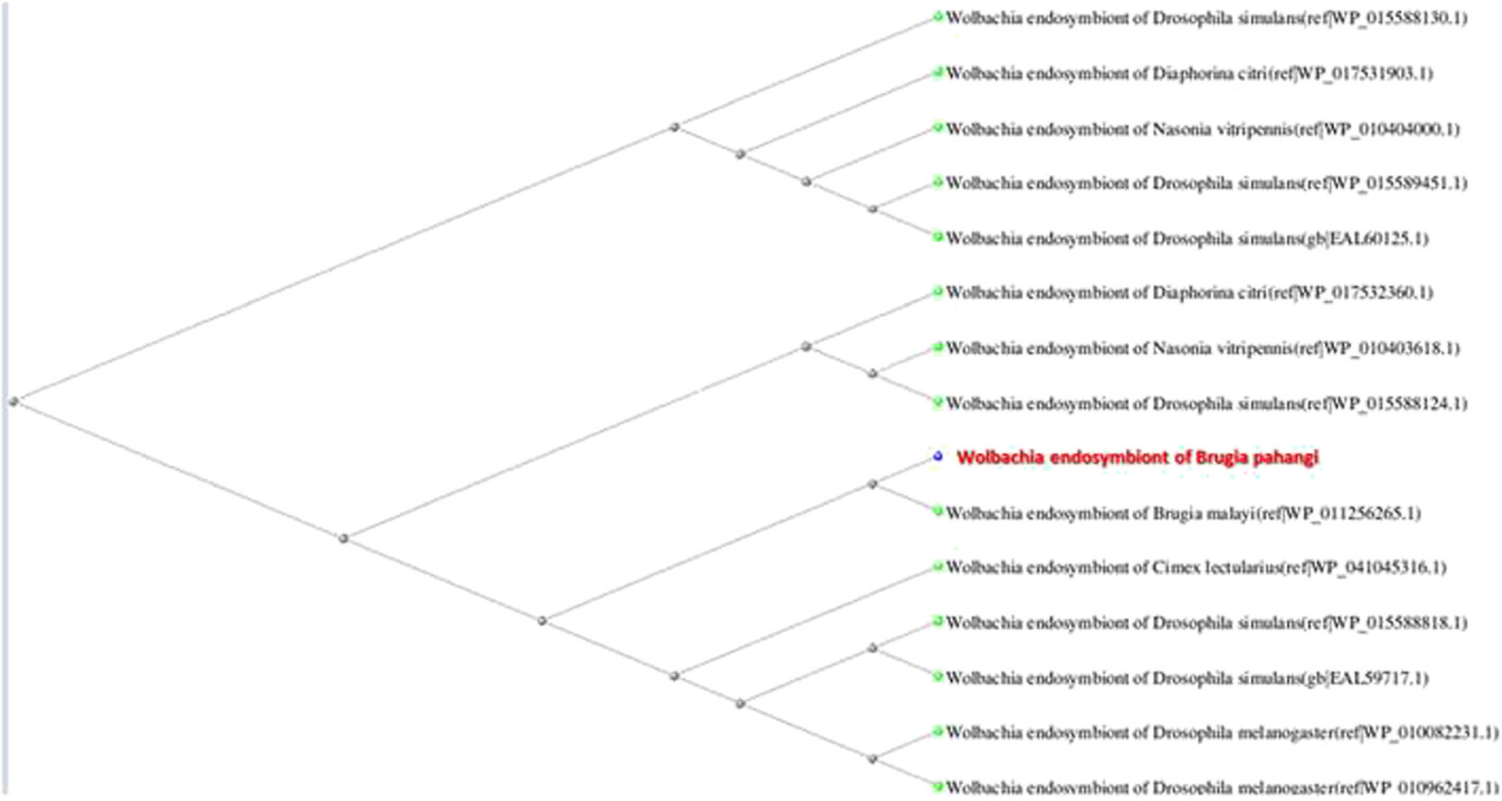
A phylogenetic tree constructed based on an ATPase protein coding gene in *Wolbachia-B. pahangi* (Brugia.Pahangi_GLEAN_10000840). The tree indicates that this gene is in the same clade with an ATPase in *Wolbachia-Brugia malayi*. The tree was generated using neighbour-joining algorithm using Kimura distance model

From the *B. pahangi* genome, 803 genes were homologuos to *B. malayi Wolbachia* genes and most of the genes were mapped to 4 distinct scaffolds (Additional file 1: Table S13), which consisted entirely of *Wolbachia* genes, implying presence of a region of genome which is not integrated within the filarial nematode. Interestingly, 44 of the *Wolbachia*-derived genes were found to be incorporated within other parts of the genome other than the aforementioned 4 distinct scaffolds, suggesting gene integration via lateral transfer between the endosymbiotic bacteria and the filarial nematode. As mentioned earlier, many *B. pahangi*-unique genes were found to be derived from its endosymbiont *Wolbachia*. Among these *Wolbachia* genes, 45 genes were mapped to metabolic pathways and features that are unique to the species, 29 genes were linked to genetic information processing pathways, 7 were involved in environmental information processing, 4 were associated with cellular processes, 7 genes were mapped to human disease pathways, and 2 genes were linked to organismal systems. In addition, 3 *Wolbachia* genes were of unknown function. We proposed that the *Wolbachia* genes may contribute to at least some of the unique characteristics of *B. pahangi*. These genes may complement *B. pahangi* cellular machinery, which further confer survival advantage to the filarial nematode within its mosquito vectors and mammalian hosts. Overall, phylogenetic analysis shows that *Wolbachia* of *B. pahangi* is phylogenetically close to *Wolbachia* of *B. malayi* (Fig. 3), which is parallel to the evolutionary relationship between the involving nematode hosts.

## Discussion

The high detection rate of core essential genes from our *B. pahangi* genome indicates that this assembly represents a substantial proportion of the entire *B. pahangi* genome. Notably, *B. pahangi* genome bears high resemblance to that of *B. malayi*. Indeed, the numbers of predicted protein-encoding genes, sizes of exons and introns as well as relative amount of repeat contents found in our *B. pahangi* genome were similar to those of *B. malayi* [46]. Besides, the high diversity of transposable elements seen in *B. pahangi* genome was similar to those encountered in genomes of other parasitic nematodes [46–49]. As expected, among the 4 species of nematodes under comparison, *B. pahangi* showed the closest genetic relationship with *B. malayi*. These findings were indeed in agreement with the phylogenetic analysis, where *B. pahangi* and *B. malayi* are originated from a more recent common ancestor relative to other nematodes recruited (Fig. 2c). The gene set in *B. pahangi* is consensus gene set from different gene predictions and homologs. In fact, 12,281 genes were predicted based on AUGUSTUS and 17,208 genes were predicted by GlimerHMM gene finder. Nevertheless, the data were made consensus to yield the best set of genes. The lower gene number (n = 9687) prediction of *B. pahangi* can be due to larger amount of repetitive regions in *B. pahangi* genome. Overall, from the whole genome sequencing, the relative amount of AT nucleotides was higher than GC nucleotides (AT bias). Interestingly, it was reported previously that the amount of coding genes in a genome is correlated to the relative amount of GC content [50]. This may explain the much lower number of genes predicted for *B. pahangi*. Nevertheless, without additional measures such as RNA sequencing, the actual causes of such discrepancy in gene numbers between these closely related *Brugia* spp. cannot be answered by this study. Meanwhile, whole genome conserved synteny analysis was conducted to identify and characterize the functionally related stretches of clustered genes between different species. Large segments exhibiting high similarity between homologous sequences from several species indicate conserved and important biological features. Therefore, the strength of evolutionary constraints and the importance of functional elements can be quantitatively measured by determining the local evolution rates in multiple alignments. The genome similarity shared between *B. pahangi* and *B. malayi* in many aspects of fundamental cellular activities may be one of the factors that enable survival and growth of *B. pahangi* in humans apart from animals as its natural host.

Genes unique to *B. pahangi* genome comprise only a small portion of the whole draft genome (~5.87 %). Nevertheless, the functions encoded by these unique genes may be responsible for the unique properties of *B. pahangi* that make it a distinct species. We identified 9 genes that mapped to metabolic pathways: glycan biosynthesis and metabolism (Brugia.Pahangi_GLEAN_10002876); cofactor and vitamin synthesis (Brugia.Pahangi_GLEAN_10005830, 10010649); amino acid metabolism (Brugia.Pahangi_GLEAN_10004798); secondary metabolite processing (Brugia.Pahangi_GLEAN_10000930); carbohydrate metabolism (Brugia.Pahangi_GLEAN_10000856, 10000857); enzyme biosynthesis (Brugia.Pahangi_GLEAN_10004319); and a yet-unclassified pathway (Brugia.Pahangi_GLEAN_10004951). In addition, 4 genes were linked to other metabolic pathways as well (Brugia.Pahangi_GLEAN_10000930, 10004798, 10000856, 10000857).

N-glycans are essential for proper protein folding in eukaryotic cells. Without the correct folding, proteins are subjected to proteolysis, which represents a quality control measure that is vital for ensuring functional efficiency of newly produced proteins. Furthermore, N-glycans are involved in cell–cell communication and protein–protein interactions. Therefore, it is not surprising that one of the unique genes in *B. pahangi* is mapped to N-glycan biosynthesis and metabolism, which is critical for survival of eukaryotic organisms and can confer unique characteristics to distinct species. There was evidence of 2 *Wolbachia* genes (Brugia.Pahangi_GLEAN_10010456, 10010462) predicted to participate in terpenoid backbone biosynthesis, which contributes to N-glycan biosynthesis and degradation, suggesting successful endosymbiotic relationship that exists between *B. pahangi* and *Wolbachia*.

Two *B. pahangi* unique genes (Brugia.Pahangi_GLEAN_10005830, 10010649) mapped to porphyrin and cobalamin (vitamin B12) metabolism. Cobalamin is involved in fundamental cellular metabolic activities, whereas porphyrin is the key ingredient for heme biosynthesis. Heme is a crucial component for several important classes of proteins (e.g., cytochromes, hemoglobins, peroxidases, and catalases), which are involved in a variety of critical biological processes, including oxidative metabolism and electron transport [51]. Heme has also been suggested to participate in embryogenesis, ecdysis, and reproduction in filarial worms [52, 53]. Moreover, heme-dependent cytochrome P450 monooxygenases are essential for biosynthesis and metabolism of ecdysteroids [54], which are critical in the development of the microfilaria of *B. pahangi* and *Dirofilaria immitis* [55, 56]. Although *B. pahangi* and *D. immitis* are unable to synthesize ecdysteroids from cholesterol [57, 58], ecdysone is efficiently metabolized by these filarial worms [57]. Clearly, heme plays vital roles in filarial worm physiology. Nevertheless, it appears that many nematodes lack complete heme biosynthesis pathways, meaning that they must salvage metabolites or intermediates from their environment or through symbiotic associations. Although these *B. pahangi* unique genes (Brugia.Pahangi_GLEAN_10005830, 10010649) are inadequate to complete the whole interactions involved in heme synthesis, we identified 4 non-integrated *Wolbachia* genes that are mapped to the same heme biosynthesis pathway (Brugia.Pahangi_GLEAN_10010415, 10010583, 10010588, 10005031). This finding suggests that the 4 genes of bacterial origin may complement heme biosynthesis in *B. pahangi*. In addition, the metabolism of some amino acids (glycine, threonine, alanine, aspartate, and glutamate) is associated with porphyrin and cobalamin synthesis. Interestingly, we also identified two non-integrated *Wolbachi*a genes that mapped to glycine and threonine metabolism (Brugia.Pahangi_GLEAN_10010377, 10005031) as well as another non-integrated *Wolbachia* gene (Brugia.Pahangi_GLEAN_10010462) mapped to alanine, aspartate and glutamate metabolism in the *B. pahangi* genome assembly. This finding might suggest that *B. pahangi* achieves enhanced metabolic efficiency through successful endosymbiotic relationship with its *Wolbachia* endosymbiont.

The ability to process and metabolize secondary metabolites can confer survival advantages to parasites. In this regard, various drug molecules, such as beta-lactams (e.g., penicillin and cephalosporin), can be metabolized by *B. pahangi* through a pathway involving one of its unique genes (Brugia.Pahangi_GLEAN_10000930). Interestingly, this *B. pahangi* gene also mapped to pathways participating in environmental information processing via two-component system (TCS) signal transduction, which is commonly found in bacteria [59]. Indeed, we identified one *Wolbachia* gene (Brugia.Pahangi_GLEAN_10010525) that is involved in TCS signalling. With TCS signalling pathway, the parasite could sense, respond, and react to chemicals encountered in its surroundings, which may help the parasite to identify its vectors and hosts. Apart from TCS system, protein kinases also play key roles in cell signaling pathways. The biosynthesis of protein kinases is mapped to one of the unique *B. pahangi* genes (Brugia.Pahangi_GLEAN_10004319). This gene may code for synthesis and regulation of distinct protein kinases that are involved in specific cellular signaling pathways in *B. pahangi*, thereby conferring differential host/vector adaptability and virulence as compared to other filarial nematodes.

We also identified another unique gene in *B. pahangi* that mapped to metabolism of cysteine and methionine (Brugia.Pahangi_GLEAN_10004798). Notably, this gene also mapped to another non-metabolic function involving a DNA repair and replication regulatory pathway. Indeed, it has been demonstrated that a high level of intracellular cysteine and homocysteine (metabolite of methionine) can affect the efficiency of the DNA repair machinery [60, 61]. Therefore, effective regulation of cysteine and methionine metabolism is essential for the maintenance of DNA integrity.

A number of unique *B. pahangi* genes were found to be involved in genetic information processing. A total of 10 unique genes were mapped to this group, revolving around 3 functions: transcription and transcription factors (Brugia.Pahangi_GLEAN_10005465, 10009860); folding, sorting and degradation (Brugia.Pahangi_GLEAN_10009906, 10005156, 10005259, 10005260, 10005261); and DNA replication/repair (Brugia.Pahangi_GLEAN_10006829, 10008037, 10004798). Eight of these genes were mapped to functions and pathways specific to gene regulation and processing, whereas the remaining 2 genes were involved in cross- KEGG class functions and pathways. Besides, 4 *B. pahangi*-specific genes (Brugia.Pahangi_GLEAN_10009906, 10005259, 10005260) are mapped to ubiquitin-related regulation. Ubiquitin is a post-translational regulatory protein that is exclusive to eukaryotic organisms. Following ubiquitination, proteins can be targeted for degradation through ubiquitin-activated proteolysis. Genes annotated to transcription factors and ubiquitin-related regulations may play roles in epigenetic regulation of *B. pahangi* as well. Indeed, epigenetic regulation via microRNAs (miRNAs) has been reported in filarial nematodes [62].

Moreover, an additional gene was found to map to a eukaryotic-exclusive sorting and folding function involving SNARE interactions during vesicular transport (Brugia.Pahangi_GLEAN_10005156). This endoplasmic reticulum-dependent function enables synthesized proteins to be exocytosed. Finally, several DNA replication and repair functions were mapped to unique *B. pahangi* genes. These include homologous recombination, non-homologous joining, DNA replication, DNA repair, DNA recombination, and chromosome repair machinery.

We identified 5 genes that mapped to environmental information processing pathways (Brugia.Pahangi_GLEAN_10005686, 10000930, 10000856, 10000857, 10009860). These genes were mapped to signal transduction pathways and linked to other KEGG pathway classes as well, including metabolism, genetic information processing, cellular processes, human disease pathways, and organismal systems. Such diverse functional mapping highlights the importance of signal transduction in the regulation of vital cellular activities. Indeed, several specific signal transduction pathways were mapped to these unique *B. pahangi* genes (e.g., mitogen activated protein kinases [MAPK] pathways, epidermal growth factor receptor [ErbB] pathways, phosphatidylinositol signaling cascades, TCS pathways, and cell adhesion molecule [CAM] ligands). These findings indicate that *B. pahangi* may employ unique strategies for cell–cell communication and cellular regulation.

According to the KEGG classification, genes mapping to “cellular processes” participate in cell growth/death, movement, communication, catabolism, and transport. A total of 8 unique genes were found to be mapped to this category. Of these, 2 were mapped to endocytosis (Brugia.Pahangi_GLEAN_10005460, 10007532), while 5 genes were linked to cell communication via tight junctions (Brugia.Pahangi_GLEAN_10009173, 10007676) or focal adhesions (Brugia.Pahangi_GLEAN_10001231, 10001865, 10009860). Both tight junctions and focal adhesions are responsible for cell anchorage. In addition, tight junctions play vital role in barrier protection and regulate signal transduction [63]. Similarly, focal adhesions also participate in biochemical signaling processes [64, 65]. There were 2 genes annotated to cell movement via actin regulation (Brugia.Pahangi_GLEAN_10001231, 10001865). In addition to regulating cell migration and division, actin is also a key component controlling muscle contraction and movement. Besides, another gene was found to be mapped to unclassified cellular processes and signalling mechanisms (Brugia.Pahangi_GLEAN_10005055).

Organismal system pathways include all cascade reactions that are associated with the immune responses, endocrine system, nervous system, circulatory system, and other organ system pathways. All 4 unique *B. pahangi* genes that coded for organismal system pathways were also linked to other functional groups, including pathways associated with human diseases, cellular processes, and environmental information processing (Brugia.Pahangi_GLEAN_10009860, 10001231, 10001865, 10005686). The mapped function pathways include the insulin and gonadotropin-releasing hormone (GnRH) signaling, as well as chemokine signaling pathways. Notably, chemokine signaling pathways are important for cellular growth, differentiation, migration, and survival, as well as regulation of reactive oxygen species (ROS) production.

Three unique genes were mapped to human disease pathway (Brugia.Pahangi_GLEAN_10009860, 10001231, 10001865). All of them coded for multiple functions spanning different pathway categories. One of these (Brugia.Pahangi_GLEAN_10009860) was mapped to pathways of genetic information processing, environmental information processing, cellular processes, and organismal systems. The other 2 were linked to functions of cellular processes and organismal systems. Of particular interest, one gene (Brugia.Pahangi_GLEAN_10009860) was linked to pathogenesis pathway related to leishmaniasis, another parasitic infection. This pathway revolves around dysregulation of MAPK cascade. The interference of MAPK cascade will affect various downstream cellular events such as DNA proliferation, gene expression and regulation, cell survival and differentiation, protein synthesis and apoptosis. Indeed, dysregulation of MAPK cascade is one of the key players in endometrial cancer, prion disease and leishmaniasis pathogeneses. For endometrial cancer, this leads to uncontrolled cell growth and proliferation [66]. In prion disease, this results in dendritic atrophy and neuronal apoptosis [67]. For leishmaniasis pathway, inhibition of host’s immune system via impairment of major histocompatibility complex (MHC) genes expression and disruption of cytokine secretion are resulted [68]. The virulence of *B. pahangi* and pathogenesis of *B. pahangi* infection may revolve around the dysregulation of host MAPK signaling cascade, be it the definitive host, vector or reservoir. Indeed, filarial worms are known for its manipulation of host’s immune system towards their benefit of survival [69]. While the hosts evolve to counter the infections, the parasites also evolve to evade host’s immune system, or down-regulate host’s immune responses to persistently exist and survive within the host, thereby sustaining the parasitic relationship with the host. As a result, a balanced point of evolution is usually preferred, where the parasites can survive within the host while causing no or minimal pathological changes to the host. For example, in lymphatic filariasis endemic areas, upregulation of auto-inhibitory cytokines such as interleukin 10 (IL-10) and transforming growth factor β (TGFβ), as well as inhibition of interleukin 12 (IL-12) secretion have been observed in asymptomatic carriers, who usually represent the majority of such population [69, 70].

Another pathogenesis-related function mapped to *B. pahangi* unique gene was the machinery of epithelial cell invasion. This machinery is derived from bacteria. Although the gene coded for this function (Brugia.Pahangi_GLEAN_10001231) is not one of those *Wolbachia*-derived genes, we believe that *B. pahangi* has acquired this gene from *Wolbachia* since the beginning of their endosymbiotic relationship. The ability of invading epithelial cell is critical for the parasite developmental stages within the mosquito vector and migration from circulation to the lymph nodes in the mammalian hosts. There are two models for the epithelial invasion machinery [71, 72]. Both mechanisms eventually lead to the manipulation of host cell’s actin cytoskeleton. Interestingly, this gene is also mapped to the modulation of actin cytoskeleton. In view of the annotations, this *B. pahangi* unique gene may also be responsible for the ability of *B. pahangi* to survive in *Armigeres subalbatus*, which is different from *B. malayi* [73].

In this project, the genome of endosymbiont *Wolbachia* was sequenced concomitantly. In fact, the presence of *Wolbachia* in *B. pahangi* worm used for genome sequencing is the major limitation of our study, where the endosymbiont bacteria should be removed prior to sequencing. Nevertheless, many studies have shown that killing of *Wolbachia* resulted in growth retardation or death of the nematode [52, 74]. Coupled with constrains of time and resources, we decided to proceed with untreated *B. pahangi* specimens and separate the *Wolbachia* genome from the filarial nematode genome after sequencing by referring to the draft genome of *Wolbachia-B. malayi* (*WBm*) [52]. Thereafter, we decided to look into these genes of *Wolbachia* origin to have a better understanding on the genetic interactions between *B. pahangi* and its endosymbiont *Wolbachia*. Here, we have selected a few interesting *Wolbachia-B. pahangi* (*WBp*) genes to discuss.

Based on analysis using KEGG database, *B. pahangi* may be benefit from metabolic processes of at least 11 amino acids, owing to unique *WBp* genes (Brugia.Pahangi_GLEAN_10010328, 10010377, 10010481, 10010510, 10005051). These amino acids included lysine, histidine, tyrosine, glycine, serine, threonine, alanine, aspartate, glutamate, arginine, and proline. In addition, one gene was annotated to metabolism of selenocompounds (Brugia.Pahangi_GLEAN_10010573). Interestingly, there were as many as 4 genes (50 % of the genes involved in amino acid biosynthesis) participating in lysine biosynthesis. Lysine is known to be an important ingredient for collagen and elastin cross-linkage [75, 76]. Nevertheless, lysine biosynthesis cascade may be incomplete even with these genes as meso-diaminopimelate (meso-DAP) decarboxylase, the enzyme required for final conversion of aspartate into lysine via meso-DAP intermediate was not found in genomes of *B. pahangi* and the endosymbiont *Wolbachia*. This is indeed similar to situations in *B. malayi* [46, 52].

Specific *Wolbachia* gene products also displayed multiple closely related functions. For instance, the gene linked to histidine and tyrosine metabolism (Brugia.Pahangi_GLEAN_10010481) was also responsible for biodegradation of xenobiotics and polycyclic aromatic hydrocarbons. Indeed, histidine is the precursor of carnosine, an antioxidant capable of neutralizing ROS derived from xenobiotics and polycyclic aromatic hydrocarbons [77]. Likewise, tyrosine has been shown to play an important role in protection against cellular oxidative stress [78]. Interestingly, genes linked to the metabolism of alanine, aspartate, glutamate, arginine, and proline are also involved in nitrogen metabolism (Brugia.Pahangi_GLEAN_10010462). Moreover, glucogenic amino acids can be precursors for gluconeogenesis [79]. Riboflavin metabolism genes were also identified in the *WBp* genes (Brugia.Pahangi_GLEAN_10010436, 10010482, 10010635, 10005045). Riboflavin (vitamin B2) is crucial for the synthesis of flavin adenine dinucleotide (FAD) and flavin mononucleotide (FMN). These factors function as important coenzymes in diverse oxidation–reduction reactions during intermediate metabolism.

In addition, unique *WBp* genes may also contribute significantly to the complexity of the environmental information and cellular processing machinery of *B. pahangi*, particularly in signal transduction. For example, a gene linked to G protein-coupled receptors (GPCRs) (Brugia.Pahangi_GLEAN_10005033) was identified. GPCRs are trans-membrane receptors that regulate cellular activity by triggering intracellular signaling cascades in response to extracellular signals, biomolecules, and environmental changes [80, 81]. There are 2 major types of signal transduction pathways involving GPCRs: cyclic adenosine monophosphate (cAMP) pathways and phosphatidylinositol signaling systems. As mentioned earlier, some *B. pahangi* unique genes were found to code for products involved in phosphatidylinositol signaling (Brugia.Pahangi_GLEAN_10000856, 10000857). Since GPCRs are involved in the regulation of various cellular activities (e.g. homeostasis and immunity), it may contribute to the specific parasite–vector relationship between *B. pahangi* and *Ar. subalbatus*, as well as the broader mammalian host range as compared to the nocturnal periodic *B. malayi*. This signal transduction machinery may enable *B. pahangi* to interact with its surroundings, including the immune cells of mosquitoes. Sensing environmental cues allows the parasite to respond by evading or impairing the mosquito’s immune system, thereby facilitating parasite survival within the mosquito vector. Similar signal transduction events may permit *B. pahangi* to cause asymptomatic infections in mammalian hosts, including humans.

As mentioned earlier, we found a number of unique genes, both from *B. pahangi* genome and *WBp* genome that are annotated to functions that may play critical role in determining vector specificity and host adaptability of *B. pahangi*. Nevertheless, it would be difficult to draw a solid conclusion on this subject based on draft genome comparison. Besides, there are 2 strains of *B. malayi*, the nocturnal periodic type that infects only humans, and the nocturnal subperiodic type that is zoonotic in nature. Genomic comparison and analysis on this subject would be more powerful if genomic data of the nocturnal subperiodic *B. malayi* and its endosymbiont *Wolbachia* are available. Nevertheless, our findings assist in narrowing the focus for further studies to seek answers for this matter.

In this project, we found that a number of genes of *Wolbachia* origin were integrated into the *B. pahangi* genome, suggestive of lateral gene transfer. Indeed, the integration of *Wolbachia* genes into their host genome has been reported previously [82, 83]. Such phenomenon was reported in *B. malayi* as well, where many of the integrated genes are pseudogenized and cannot be expressed by the nematode [52]. Nevertheless, based on sequence alignment alone, it would be difficult to determine whether an intact, non-degenerated integration is pseudogenized. Therefore, more studies are required to test the functionality of these integrated genes in *B. pahangi*.

## Conclusions

As expected, the genome of *B. pahangi* shares high similarity with that of *B. malayi*. Nevertheless, there are distinct features in *B. pahangi* genome that may confer unique physiological and pathological differences, as well as features that may influence its interaction with vectors and mammalian hosts. The unique genes found in *B. pahangi* genome may serve as reference to study biological and virulence differences among different filarial nematodes in future. Moreover, study of the *B. pahangi* genome has illuminated the genetic interaction between *B. pahangi* and its endosymbiont *Wolbachia*. Indeed, it appears that *Wolbachia*-derived genes contribute and complement the content and uniqueness of the *B. pahangi* genome. Such interaction between *B. pahangi* and its endosymbiont bacteria at genetic level may serve as additional reference to better understandings on cellular biology of other filarial nematodes.

## Additional file

Additional file 1: Table S1. Statistic of pre-filter data. A total of 24 GB of raw data were generated and used for assembly with total sequence depth of 214.29 and total physical depth of 3203.63. **Table S2.** Statistic post filtering. A total of 18.76 GB data were generated after filtering process with total sequence depth of 167.5 and total physical depth of 2290.07. **Table S3.** Transposable elements (TEs) content in the Assembled *B. pahangi* Genome. The highest TEs predicted at the DNA level was found by RepBase (0.75 %). **Table S4.** General statistics of predicted protein-coding genes. Three de novo based methods used: AUGUSTUS, SNAP and GLIMMERHMM. **Table S5.** Statistics of function annotation. The method for functional annotation is divided into two types that are automated and manual. The automated method, TrEMBL database shows the highest number of annotation among all methods used with percentage of 96.64 % while InterPro database produces the most number of annotations with percentage of 71.32 % among manually curated databases. **Table S6.** *B. pahangi* genes mapped to Swissprot via blast. **Table S7.** *B. pahangi* genes with intrepro annotations. **Table S8.** *B. pahangi* genes with Gene ontology annotations. **Table S9.** *B. pahangi* genes mapped to KEGG via blast. **Table S10.** List of 569 *B. pahangi* unique genes. **Table S11.** The 403 *Wolbachia* genes in *B. pahangi* unique genes. **Table S12.** The 26 *B. Pahangi* unique proteins with their respective KEGG pathway class. **Table S13.** The 803 *Wolbachia* genes in *B. pahangi* genome. **Table S14.** General statistics of repeats in genome. From the table, TRF shows the biggest repeat size of 2.9 Mbp, which represent 3.34 % of the genome size. (XLSX 2292 kb)
